# Characterization, identification and evaluation of a set of wheat-*Aegilops comosa* chromosome lines

**DOI:** 10.1038/s41598-019-41219-9

**Published:** 2019-03-18

**Authors:** Cheng Liu, Wenping Gong, Ran Han, Jun Guo, Guangrong Li, Haosheng Li, Jianmin Song, Aifeng Liu, Xinyou Cao, Shengnan Zhai, Dungong Cheng, Genying Li, Zhendong Zhao, Zujun Yang, Jianjun Liu, Stephen M. Reader

**Affiliations:** 1 Crop Research Institute, Shandong Academy of Agricultural Sciences/Key Laboratory of Wheat Biology and Genetic Improvement in the North Yellow & Huai River Valley, Ministry of Agriculture/National Engineering Laboratory for Wheat & Maize, Jinan, 250100 China; 2 Colloge of Life Science, Shandong Normal University, Jinan, 250014 China; 3 School of Life Science and Technology, University of Electronic Science and Technology of China, Chengdu, 610054 China; 4 John Innes Centre, Norwich Research Park, Colney, Norwich, NR4 7UH UK

## Abstract

This study characterized and evaluated a set of wheat-*Aegilops comosa* introgression lines, including six additions and one substitution. A total of 47 PLUG markers and a set of cytogenetic markers specific for *Ae*. *comosa* chromosomes were established after screening 526 PLUG primer pairs and performing FISH using oligonucleotides as probes. Marker analysis confirmed that these lines were wheat-*Ae*. *comosa* 2M–7M addition lines and a 6M(6A) substitution line. The molecular and cytogenetic markers developed herein could be used to trace *Ae*. *comosa* chromatin in wheat background. In order to evaluate the breeding value of the material, disease resistance tests and agronomical trait investigations were carried out on these alien chromosome introgression lines. Disease resistance tests showed that chromosomes 2M and 7M of *Ae*. *comosa* might harbor new stripe rust and powdery mildew resistance genes, respectively, therefore, they could be used as resistance sources for wheat breeding. Investigations into agronomical traits showed that all chromosomes 2M to 7M had detrimental effects on the agronomic performance of wheat, therefore, the selection of plants with relatively negative effects should be avoided when inducing wheat-*A*. *comosa* chromosome translocations using chromosome engineering procedures.

## Introduction

*Aegilops comosa* Sm. in Sibth. et Sm. (syn. *Triticum comosum* (Sm. in Sibth. et Sm.) K. Richt.) is an annual diploid species (2*n* = 2x = 14, genome MM) with narrowly cylindrical spikes, slender glumes, parallel veins and three awns, mainly endemic to coastal regions of the former Yugoslavia, Albania, and coastal and inland Greece^1^. *Ae*. *comosa* has been found to be resistant to wheat stripe rust (*Puccinia striiformis* Westend)^2^, leaf rust (*P*. *recondita* Roberge ex Desmaz. f. sp. *tritici*) and powdery mildew (*Blumeria graminis* f. sp. *tritici*)^3^, stem rust (*Puccinia graminis* f. sp. *tritici*)^4,5^, cereal cyst nematode (*Heterodera avenae* Wollenweber)^6^, hessian fly (*Mayetiola destructor* (Say) and greenbug (*Schizaphis graminum* (Rondani)^3^. Moreover, *Ae*. *comosa* has salt tolerance^7^. Therefore, *Ae*. *comosa* is potentially an excellent gene source for wheat improvement.

The creation of wheat-*Ae*. *comosa* amphiploids, addition, substitution and translocation lines are the first steps in the long process of transferring desirable genes from *Ae*. *comosa* to wheat. Riley *et al*. produced and identified a wheat-*Ae*. *comosa* 2M addition line^2^. Moreover, 2D-2M translocations^2,8^ and 2A-2M translocations^5,8^ were also developed. Weng *et al*. synthesized a *T*. *persicum*-*Ae*. *comosa* amphiploid and six addition lines^9^, but the homoeologous groups of the *Ae*. *comosa* chromosomes in these additions were not identified. Hereafter, Weng *et al*. created a wheat-*Ae*. *comosa* 4M(4D) substitution line^10^. Recently, Jia (2016) synthesized a wheat-*Ae*. *comosa* amphiploid and obtained several wheat-*Ae*. *comosa* derivatives^11^. Besides the wheat-*Ae*. *comosa* chromosome lines mentioned above, no other references have been found reporting the development of wheat-*Ae*. *comosa* germplasm.

Miller’s research group at John Innes Centre, UK, developed six wheat-*Ae*. *comosa* additions and one substitution. These lines were primarily identified by studying mitotic chromosomes used the classic method based on marker loci^12^ for determining the homoeology of chromosomes within the Triticeae tribe. These lines were named as wheat-*Ae*. *comosa* 2?M-7?M additions (? indicates the homoeologous group of the M genome chromosomes in wheat background is a speculation) and a 6?M (6A) substitution. In this current research, PCR-based landmark unique gene (PLUG) markers were developed to confirm the previous tentative chromosomal identifications. In order to facilitate any future utilization of wheat-*comosa* chromosome translocations for wheat breeding programs, cytogenetic markers specific for *Ae*. *comosa* chromosomes were developed to assist in identifying *Ae*. *comosa* chromosomes in a wheat background. In addition, the levels of disease resistance and agronomical characteristics of the wheat-*Ae*. *comosa* 2M–7M additions and the 6M(6A) substitution were also investigated to provide useful information for the possible future development of wheat-*Ae*. *comosa* translocations or homoeologous recombinants.

## Results

### Identification of wheat-*Ae*. *comosa* chromosome lines using PLUG markers

**Figure 1:**
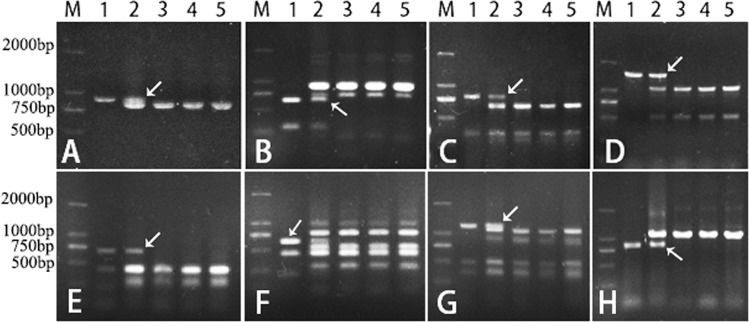
PCR patterns of primer pairs TNAC1204 (**A**), TNAC1137 (**B**), TNAC1329 (**C**), TNAC1331 (**D**), TNAC1737 (**E**), TNAC1740 (**F**), TNAC1800 (**G**) and TNAC1924 (**H**). Lane M indicates Marker DM2000. Lanes 1–5 in (**A–H**) are *Ae*. *comosa*, *T*. *turgidum-Ae*. *comosa* amphiploid, CS, JM22 and JN17, respectively.

Molecular markers developed based on EST primers had been widely used in identifying chromosome homoeologous groups in wheat-interspecific crosses^13,14^. In this research, a total of 526 PLUG primer pairs were screened using *Ae*. *comosa*, the *T*. *turgidum-Ae*. *comosa* amphiploid, CS, JM22 and JN17 as material to develop chromosome specific markers for *Ae*. *comosa*. As a result, fifty-four primer pairs were found which could generate polymorphism(s) among the material tested (Table 1). Among these fifty-four primer pairs, five, seven, eighteen, three, two, eight and eleven belonged to chromosome homoeologous groups 1 to 7, respectively, and they were *Ae*. *comosa*-specific. The PCR patterns of primer pairs TNAC1204, TNAC1137, TNAC1329, TNAC1331, TNAC1737, TNAC1740, TNAC1800 and TNAC1924 were showed in Fig. 1.

**Table 1:** PLUG primer pairs screened to identify specific markers of *Ae*. *comosa* chromosomes.

Chromosome homoeologous group	Number of PLUG primer pairs	Number of polymorphic primer pairs	Number of polymorphic markers which could be located on *Ae*. *comosa* chromosomes	% polymorphism
Group 1*	57	5	5	8.8%
Group 2	67	7	7	10.4%
Group 3	85	18	16	18.8%
Group 4	71	3	1	1.4%
Group 5	78	2	1	1.3%
Group 6	59	8	7	11.9%
Group 7	109	11	10	9.2%
Total	526	54	47	8.9%

*Data are collected from CS-*Ae*. *geniculata* 1M^g^ addition but not the CS-*Ae*. *comosa* 1M addition.

PCR using the fifty-four PLUG primers mentioned above was also performed on the tentatively named CS-*Ae*. *comosa* 2M–7M additions and the 6M(6A) substitution to identify the homoeologous groups for each of the *Ae*. *comosa* chromosomes. The M genome of *Ae*. *comosa* was the donor of the M^g^ genome of *Ae*. *geniculata*^15^. Therefore, CS-*Ae*. *geniculata* 1M^g^, 2M^g^ and 7M^g^ addition lines were also introduced into the current molecular experiment (the 3M^g^–6M^g^ addition lines are not available). The PCR results suggested that a total forty-seven markers could be located onto the M or M^g^ chromosomes (Tables 1 and 2). Among these markers, five could be located onto *Ae*. *geniculata* chromosome 1M^g^, however, they could not produce target bands from the seven CS-*Ae*. *comosa* lines, indicating that these lines do not possess chromosome 1M of *Ae*. *comosa*. Seven (only three could located on chromosome 2M^g^ of *Ae*. *geniculata*), sixteen, one, one, seven and ten (only five could located on chromosome 7M^g^ of *Ae*. *geniculata*) markers could be located onto the tentatively named chromosomes 2M to 7M of *Ae*. *comosa* (Table 2), respectively, confirming that the chromosomes in these lines were correctly identified as 2M to 7M.

**Table 2:** Markers specific for *Ae*. *comosa* chromosomes developed by the current study.

No.	Primer	Primer sequence (5′-3′)	Wheat chromosomal location*	Location on M or M^g^ chromosome	Enzyme used	Product size (bp)
1	1017	F:ACAGCCAAGGGTATCACTTCC	1B	1M^g^	*Taq*I	1250
		R:TCAAGAAAGCGCTTGTCAAAT	1D			
2	1031	F:GAGATGGAAGCGACATCTCTG	1B	1M^g^	*Taq*I	350
		R:CACAGCCCGTTGTCTGTACTT				
3	1073	F:CTGCTCGAGCTCTTCCAATTC	1B	1M^g^	*Taq*I	730
		R:CGCCAAGCAAATGAAAGTAAT	1D			
4	1089	F:CGTATGGGAAGATCACAGACC	1B	1M^g^	*Taq*I	380
		R:TGGTTTCGCATACACATCAAA	1D			
5	1079	F:CACTGTGAAGACCATGATTGC	1B	1M^g^	*Hae*III	350
		R:TCATCAGGTGGATCAACTTCC	1D			
6	1098	F:AGCGTCAGTCATCTCAGTGCT	2A	2M	*Hae*III	220
		R:CCATCTCCCTCTCCTGGAA	2B			
7	1138	F:CAAACTCCAGCACAGGGATAC	2A	2M	—	500
		R:CATCAAACAGTCCATGAGATGC	2D			
8	1204	F:GAGAGGAATGCGTGAAGTTTG	2AL4-0.27-0.77	2M	—	850
		R:AGACCATCTTTCCGGTCTTTG	2BL7-0.50-0.58			
			2DL10-0.49-0.58			
9	1137	F:GCTGAATCACTCAACCATTCC	2AL4-0.27-0.77	2M	*Taq*I	680
		R:TGCTCGCGCTCTACTTCAC	2BL4-0.65-0.89	2M^g^		
			2DL9-0.76-0.94			
10	1239	F:TGGAAACTCTGCTGCATCTCT	2B	2M	*Taq*I	500
		R:GAATATCTGGGCTCGCTTGTA		2M^g^		
11	1206	F:ACCTCTACACCAGAGCAGTCG	2A	2M	—	1200
		R:CCGAACACCTTGGACACC	2B			
12	1102	F:GGAGAGGTGAAGGACCAACTC	2AS5-0.78-1.00	2M	*Taq*I	1000
		R:CCTTGCAGCGTAGTGAGATTT	2BS3-0.84-1.00	2M^g^		
			2DS5-0.47-1.00			
13	1294	F:CGGAAACTTTAGCCTTCTGCT	3AS4-0.45-1.00	3M	*Hae*III	760
		R:GTCGTGTCAGATGCTTTGGAT	3BS9-0.57-0.78	5M		
			3DS4-0.59-1.00			
14	1254	F:ATTGATTTCAGCCCTGGAGTT	3A	3M	*Taq*I	900
		R:CTACTGCACGCACCAGAAGTT	3B	5M		
15	1244	F:TCCTGTGGTAGTTCGCTTGAC	3A	3M	—	1100
		R:CAAATGGTTAAGCCGGAATTT	3B			
16	1262	F:AGCGTCAGAATGACAGACACC	3D	3M	—	1100
		R:CTTTGTATGCCTCGGAGATCA				
17	1348	F:GCTCTCAATTGCAGGTGTTTG	3B	3M	—	950
		R:AGCGGAATCAAAGTTGAAGGT	3D			
18	1314	F:AGGCTAAGGTGACGAGCAAA	3AS4-0.45-1.00	3M	*Taq*I	780
		R:TCATCATCAAAGCATTCACCA	3BS1-0.33-0.57			
			3DS10-0.31-0.44			
19	1296	F:GCATCCTGTCCCTCATCAC	3AS4-0.45-1.00	3M	*Hae*III	1200
		R:TCGAGGTCTCTAGACCAATGC	3BS9-0.57-0.78			850
			3DS4-0.59-1.00			
20	1318	F:CATGTTCAGTGGCCTACCAGT	3A	3M	*Hae*III	550
		R:CGCATACTTCAAAGTCCAACG	3D			
21	1643	F:ACGTGGAACACAAAGCAGGTA	3A	3M	*Hae*III	730
		R:CTTCACCTCCACTTGCACATT	3B			
22	1327	F:AGAGACCCAAGCAGGATGATT	3A	3M	*Taq*I	550
		R:TGCTCCACTATCACACGACTC				
23	1329	F:CACATCCTCGTTGCTTACCAT	3D	3M	*Taq*I	800
		R:TTGGTGATGATGACCTCAAGC				
24	1331	F:GACGCGTATGTGGAGACATTT	3B	3M	*Taq*I	1700
		R:AGCTCCACCAGAGATACGTCA				
25	1349	F:CTGCCCACATAACCCTTCTG	3D	3M	*Taq*I	800
		R:AGGAGACAGGCCACCGTGA				
26	1350	F:AGCAGCAAAGGTTAGTGCGTA	3A 3B	3M	*Taq*I	1400
		R:AGAATCTGCCAGGCTGAGAAT				
27	1341	F:GTTGAAGCCTACATGCCACAC	3AL1-0.26-0.42	3M	—	600
		R:TAGCATGGGCTCCTAACATTG	C-3BL2-0.22			
			C-3DL1-0.23			
28	1364	F:CGTCAGGCTCAGGGTGTC	3AL4-0.61-0.78	3M	*Taq*I	800
		R:AAAGAGCCTCTGTCTCTCAGG	3BL10-0.50-0.63			
			3DL1-0.23-0.81			
29	1396	F:TACCGCTTCCGCTTCTTC	4B	4M	*Taq*I	1000
		R:TGAAATGGAAAGGGAATGTCA	4D	7M		
30	1496	F:TGGTGCTTCTTCGACTTCTTG	5B	4M	*Taq*I	800
		R:GCTACAACCCGGCACTCAT		5M		750
				7M		
31	1702	F:CATGGAAAGGTTGACAAGGAA	C-6AL4-0.55	6M	*Taq*I	1100
		R:CTGGATGTTCCATTTCTGCTC	C-6BL3-0.36			
			6DL6-0.29-0.47			
32	1737	F:CCCGCTGTAGACATCCTCTCT	6B	6M	*Taq*I	730
		R:GGACGATGGTCGGAATCTT	6D			
33	1740	F:CGGAAGTGCTCGATTGTATCT	6AL7-0.88-0.90	6M	*Taq*I	650
		R:GCGGGTTTCTTCTCAACCTT	6BL5-0.40-0.66			
			6DL6-0.29-0.47			
34	1745	F:AGAACTCAGATGCAGGCTCAA	6B	6M	*Taq*I	780
		R:AACAAGATGGCGAGGAAGAAC	6D			
35	1752	F:GTAGACGATGTCGAGGAGCAT	6AL8-0.90-1.00	6M	*Taq*I	780
		R:CTTCACCAATTTCTCCCATGA	6BL1-0.70-1.00			
			6DL11-0.74-0.80			
36	1740	F:CGGAAGTGCTCGATTGTATCT	6AL7-0.88-0.90	6M	—	1100
		R:GCGGGTTTCTTCTCAACCTT	6BL5-0.40-0.66			
			6DL6-0.29-0.47			
37	1751	F:CTTCCTTTGCTTGTGATCCTG	6AL8-0.90-1.00	6M	—	650
		R:GCCTGAGGACTTGAAGTGGTA	6BL1-0.70-1.00			
			6DL12-0.68-0.74			
38	1800	F:AACCATGCATCCGGTGAAC	7A	7M	*Taq*I	1200
		R:CTCATCATCGCTTCGCTCTT	7D			
39	1920	F:CTGTGACGCCCTAGAATCTGA	7D	7M	*Taq*I	800
		R:CAAGTCGACGGTACTCTCTGG				700
40	1915	F:AGCTCCAGAAAGAGCAGCAG	7A	7M	*Taq*I	720
		R:TTTCTCGACATAACGGTATGGAT		7M^g^		
41	1924	F:TAGCTTTGGAACGATGTGTGG	7A	7M	*Hae*III	770
		R:TGTGGAGCAGTGCTGTTTATG	7D	7M^g^		
42	1897	F:CAAGGAGTCGCTAAGAGATGC	7A	7M	*Taq*I	650
		R:ATTGGATATGTGCCCGATAAA	7B			
43	1823	F:TTGCTGTTCCACACGTTGATA	7B	4M	*Taq*I	720
		R:ATCGTCGAGTACGCCAACA	7D	7M		
44	1888	F:AGGGATGTGTTGGAGCTGTTA	C-7AL1-0.39	4M	*Taq*I	1400
		R:CACAGTGACCTTCTGCTCCTT	7BL2-0.38-0.63	7M,7M^g^		
			7DL5-0.30-0.61			
45	1889	F:ACCTGTTGCAAAGCCTTGAT	7B	4M	*Taq*I	750
		R:TGACCCAGAGTTGTTAGAAGC		7M		
46	1902	F:AATACCAGGTCCTCCAACTTT	7A	4M	*Taq*I	630
		R:TGGAATCGCTGAGAAAGAATG	7D	7M, 7M^g^		
47	1884	F:TATTTGACATGTTGGGCCTCT	7B	4M	—	700
		R:GGAGAAATCTGTTTGCGTTGT		7M, 7M^g^		

*Information of wheat chromosomal locations is according to Ishikawa *et al*. (2007).

**Figure 2:**
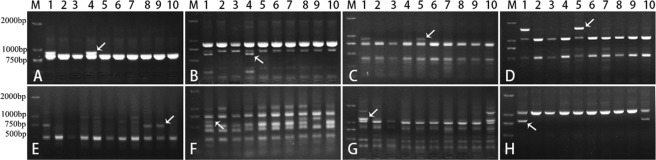
PCR patterns of primer pair TNAC1204 (**A**), TNAC1137 (**B**), TNAC1329 (**C**), TNAC1331 (**D**), TNAC1737 (**E**), TNAC1740 (**F**), TNAC1800 (**G**) and TNAC1924 (**H**). Lane M indicates Marker DM2000. Lanes 1–10 in (**A,H**) are *T*. *turgidum-Ae*. *comosa* amphiploid, CS, CS-*Ae*. *geniculata* 1M^g^ addition, CS-*Ae*. *comosa* 2M–6M additions, CS-*Ae*. *comosa* 6M(6A) substitution and CS-*Ae*. *comosa* 7M addition, respectively.

The percentage of polymorphic markers generated for chromosome homoeologous groups 1 to 7 of *Ae*. *comosa* by PLUG primers ranged from 1.3% to 18.8% (percentage data of group 1 are from *Ae*. *geniculata* due to no CS-*Ae*. *comosa* 1M addition being available) with an average of 8.8% (Table 1). The marker localization results of primer pairs TNAC1204, TNAC1137, TNAC1329, TNAC1331, TNAC1737, TNAC1740, TNAC1800 and TNAC1924 are shown in Fig. 2.

### FISH patterns of *Ae*. *comosa* chromosomes using oligonucleotides as probes

**Figure 3:**
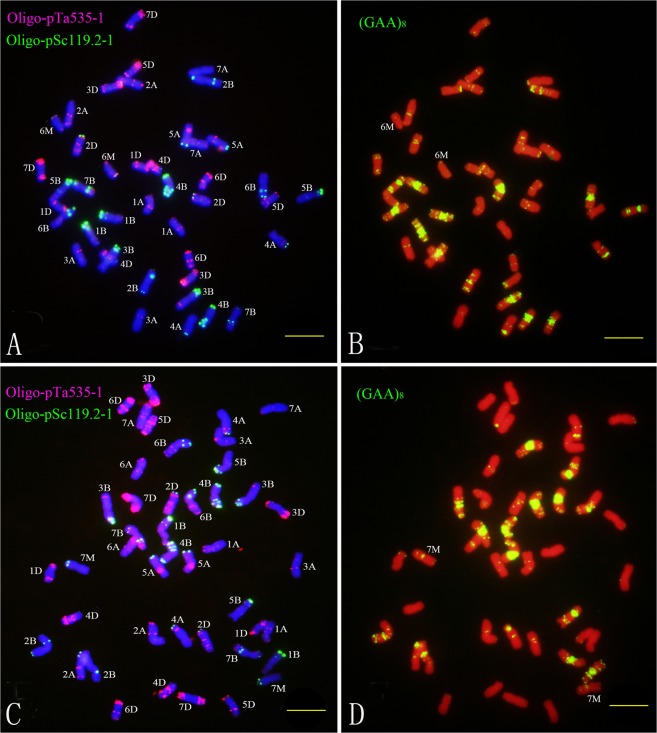
FISH using Oligo-nucleotides as probes on the CS-*Ae*. *comosa* 6M(6A) substitution (**A**,**B**) and the 7M addition (**C**,**D**). (**A,C**) are double-color FISH patterns using Oligo-pTa535–1 (red) and Oligo-pSc119.2-1 (green) as probes; (**B,D**) are FISH patterns using (GAA)_8_ (yellow) as a probe after washing off the double-color FISH signals. Bar indicates 10 µm.

Chromosome specific molecular markers of wheat alien species are useful tools for screening, identifying and utilizing wheat-alien species germplasm. Our previous study found that the sequential double-color FISH and single-color FISH could be used to identify all chromosomes of *Ae*. *uniaristata*^16,17^, *Ae*. *mutica*^13^ and *Hordeum chilense*^18^ simultaneously, as well as all 42 wheat chromosomes. Therefore, we suspected that this sequential FISH might be also useful to identify *Ae*. *comosa* chromosomes introduced into wheat. Subsequently, this FISH procedure was performed on mitotic metaphase chromosomes of the *T*. *turgidum-Ae*. *comosa* amphiploid and the CS-*Ae*. *comosa* 2M–7M additions. The results showed that all wheat and *Ae*. *comosa* chromosomes could be recognized simultaneously. The FISH patterns of CS-*Ae*. *comosa* 6M(6A) substitution and 7M addition are shown in Fig. 3.

**Figure 4:**
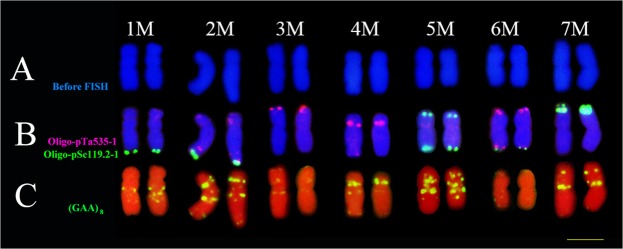
Standard FISH pattern of *Ae*. *comosa* chromosomes using Oligo-nucleotides as probes. Row (**A**) chromosomes before FISH; Row (**B**) double-color FISH patterns using Oligo-pTa535-1 (red) and Oligo-pSc119.2-1 (green) as probes; Row (**C**): FISH patterns using (GAA)_8_ (yellow) as probe after washing off the double-color FISH signals.

Double-color FISH showed that probes Oligo-pTa535-1 and Oligo-pSc119.2-1 mainly hybridized onto the terminal or subterminal regions of *Ae*. *comosa* chromosomes, while probe (GAA)_8_ mainly hybridized to centromeric or subtelomeric regions of the *Ae*. *comosa* chromosomes (Fig. 4). The satellited chromosome 1M had Oligo-pSc119.2-1 signals on terminal regions of long arms. Chromosome 2M had Oligo-pSc119.2-1 signals on terminal regions of long arms, and had Oligo-pTa535-1 signals on subterminal regions of long arm and terminal regions of the short arms. Chromosome 3M had Oligo-pTa535-1 signals on terminal regions of short arm. Chromosome 4M had Oligo-pTa535-1 signals on centromeric regions. Chromosome 5M had Oligo-pSc119.2-1 signals on both terminal regions of long and short arms. Chromosome 6M had both Oligo-pSc119.2-1 and Oligo-pTa535-1 signals on terminal regions of long arms, and had Oligo-pTa535-1 signals on terminal regions of short arms. Chromosome 7M had very strong Oligo-pSc119.2-1 signals on terminal regions of short arms. The FISH patterns of probe (GAA)_8_ also produced different signals on seven pairs of *Ae*. *comosa* chromosomes as shown in Fig. 4.

### Spike and grain characters of wheat-*Ae*. *comosa* chromosome lines

**Figure 5:**
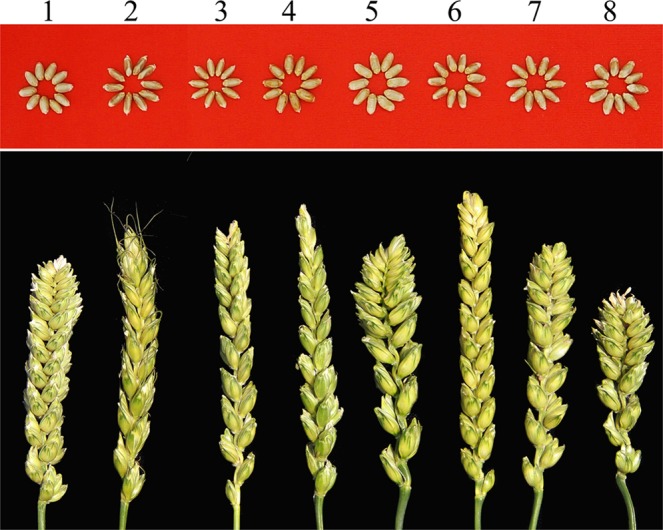
Spike and grain morphologies of wheat-*Ae*. *comosa* chromosome lines. Grain and spikes from left to right are CS, CS-*Ae*. *comosa* 2M–7M addition lines and the 6M(6A) substitution line. All the spikes and grain are collected from Jinan, Shandong Province.

Spike morphologies of the CS-*Ae*. *comosa* 2M–7M addition lines and the 6M(6A) substitution line all varied compared to that of CS (Fig. 5). Spikes of the CS-*Ae*. *comosa* 2M addition had short awns, and the lower inter-spikelet segments of the heads of CS-*Ae*. *comosa* 3M, 5M, 7M additions and the 6M(6A) substitution were more elongated compared to CS. The CS-*Ae*. *comosa* 6M addition line showed slightly elongated spikelets and overall longer spikes than CS. The CS-*Ae*. *comosa* 6M(6A) substitution showed shorter spikes and fewer spikelets compared to CS.

Grain morphologies of the CS-*Ae*. *comosa* 4M, 6M, 7M addition lines and the 6M(6A) substitution line were similar to that of CS (Fig. 5), while the CS-*Ae*. *comosa* 2M and 3M additions showed slender grains compared to CS, and 5M addition showed slightly larger grains compared to CS. CS-*Ae*. *comosa* 2M (brown) and 4M (dark yellow) additions had darker seed coat colors than the control CS, while the CS-*Ae*. *comosa* 5M addition had a lighter seed coat color than the control CS.

### Disease resistance tests of wheat-*Ae*. *comosa* chromosome lines

In this current research, wheat stripe rust, leaf rust, stem rust and powdery mildew resistance of CS, the CS-*Ae*. *comosa* 2M–7M addition lines and the 6M(6A) substitution line were tested. The results showed that all the material tested were moderately to highly susceptible to leaf rust and stem rust (Table 1). The CS-*Ae*. *comosa* 2M addition was nearly immune to stripe rust while CS and other CS-*Ae*. *comosa* chromosome lines tested were all highly susceptible to stripe rust (Table 1), suggesting that chromosome 2M of *Ae*. *comosa* carries stripe rust resistant gene(s). The CS-*Ae*. *comosa* 7M addition line was nearly immune to powdery mildew while CS and all other CS-*Ae*. *comosa* chromosome lines tested were highly susceptible to powdery mildew (Table 3), indicating that chromosome 7M of *Ae*. *comosa* carries powdery mildew resistant gene(s).

**Table 3:** Stripe rust, leaf rust, stem rust and powdery mildew infection types of CS-*Ae*.

Genotype	Stripe rust	Leaf rust	Stem rust	Powdery mildew
CS-*Ae*. *comosa* 2M addition	;	3	3	4
CS-*Ae*. *comosa* 3M addition	4	4	4	4
CS-*Ae*. *comosa* 4M addition	4	3	4	3
CS-*Ae*. *comosa* 5M addition	4	4	4	4
CS-*Ae*. *comosa* 6M addition	4	3	4	4
CS-*Ae*. *comosa* 7M addition	4	3	3	;
CS-*Ae*. *comosa* 6M(6A) substitution	4	3	4	4
CS	4	4	4	4

*comosa* chromosome lines.; denotes high resistance, 3 and 4 rating denote susceptibility.

### Agronomic trait investigation of wheat-*Ae*. *comosa* chromosome lines

**Figure 6:**
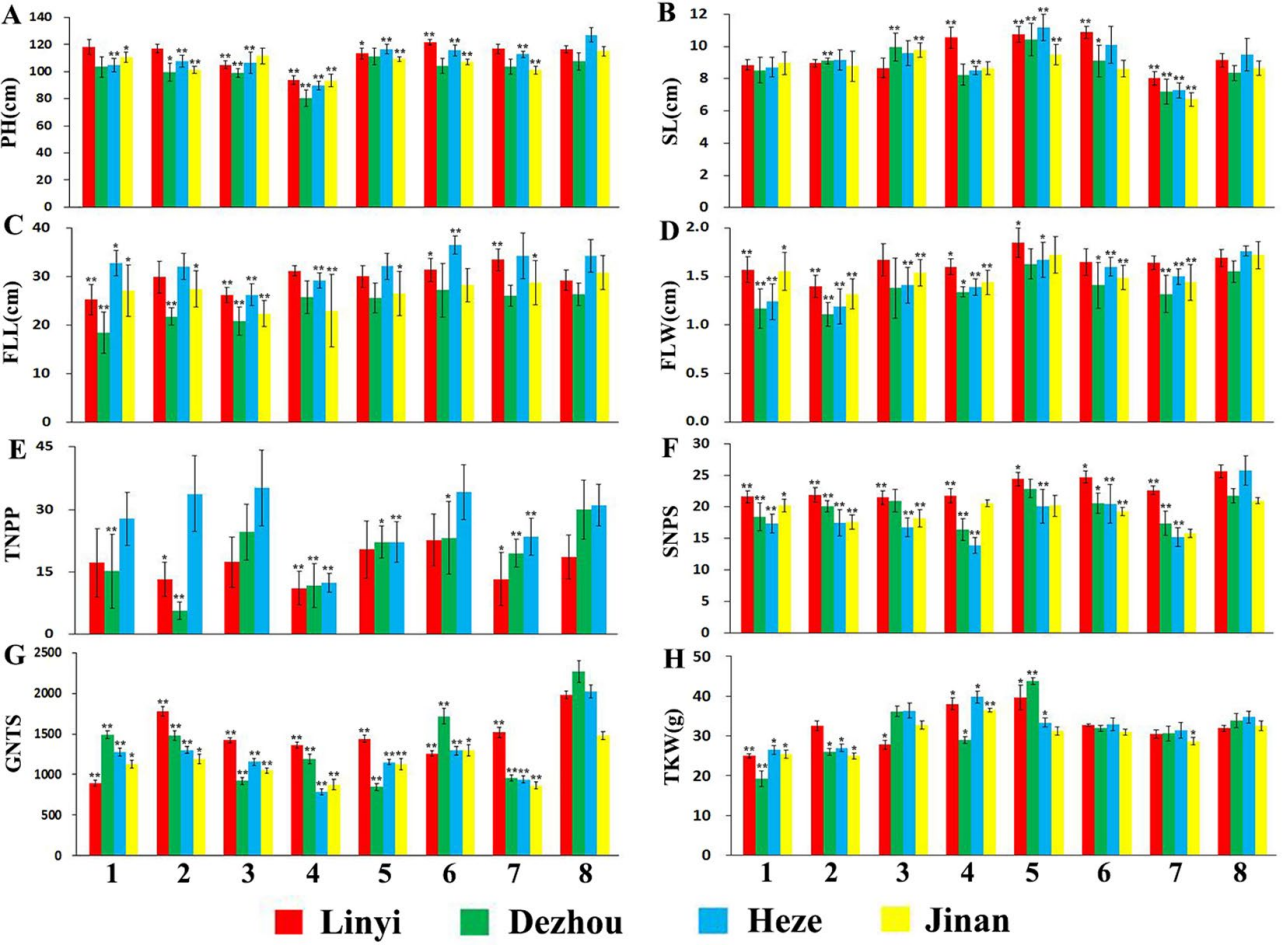
Agronomical traits investigation result of the material tested Data of tiller number from Jinan City were not obtained due to crop rotation. PH, SL, FLL, FLW, TNPP, SNPS, GNTS and TKW are the abbreviations of plant height, spike length, flag leaf length, flag leaf width, tiller number per plant, spikelet number per spike, grain number of thirty spikes and thousand kernel weight, respectively. 1 represents CS, 2–8 represents CS-*Ae*. *comosa* 2M–7M addition lines and 6M(6A) substitution line. *significant at *P* < 0.05 by comparing relative data to that of CS; **significant at *P* < 0.01 by comparing relative data to that of CS. Bar represents standard deviation.

Plant height, spike length, flag leaf length and width as well as other four agronomic traits of CS, the CS-*Ae*. *comosa* 2M–7M addition lines and the 6M(6A) substitution, were studied. The results showed that generally a detrimental effect occurred when different chromosome pairs of *Ae*. *comosa* were introduced into wheat. The introduction of chromosome 2M of *Ae*. *comosa* into CS appeared to reduce plant height (Fig. 6A) and also had a negative influence on flag leaf width, spikelet number/spike, grain number/30 spikes and thousand grain weight (Fig. 6D,F–H). Chromosome 3M of *Ae*. *comosa* introduced into CS showed a negative impact on flag leaf width, spikelet number/spike and grain number/30 spikes (Fig. 6D,F,G). Chromosome 4M of *Ae*. *comosa* introduced into CS appeared to reduce plant height (Fig. 6A) and also had a negative influence on flag leaf length, spikelet number/spike, grain number/30 spikes (Fig. 6C,F,G). The introduction of chromosome 5M of *Ae*. *comosa* into CS appeared to reduce plant height (Fig. 6A) and also had a negative impact on flag leaf width, tiller number/plant, spikelet number/spike and grain number/30 spikes (Fig. 6D–G). Chromosomes 6M and 7M of *Ae*. *comosa* introduced into CS both showed a negative influences on spikelet number/spike, grain number/30 spikes (Fig. 6F,G), while when a pair of wheat 6A chromosome was substituted by a pair of 6M chromosomes of *Ae*. *comosa*, a negative impact occurred on flag leaf width, tiller number/plant and grain number/30 spikes (Fig. 6D,E,G).

## Discussion

### Development of molecular and cytogenetic markers specific for *Ae*. *comosa* chromosomes and their utilization

Morphological studies^12,19,20^, cytogenetic markers^13,21^, biochemical markers^20,22^ and molecular markers^13,14^ have previously been used to determine chromosome homoeologous groups of alien-derived chromosomes in wheat backgrounds. All the methods mentioned above have been used in the identification of wheat-*Ae*. *comosa* germplasm^4,8–11^. Among these methods, molecular marker development is one of the easiest, quickest and cheapest approaches. However, the molecular markers specific for *Ae*. *comosa* chromosomes are currently limited in number. In this research, we developed 47 PLUG markers in order to identify the homoeologous group of each of the *Ae*. *comosa* chromosomes in six wheat-*Ae*. *comosa* addition lines and one substitution line. The results were consistent with the method^12^ (mitosis combined with morphologies etc.) that Miller and Reader used (material identification result not published), indicating that both methods for determining the homoeology of chromosomes within the Triticeae were accurate. Therefore, the EST-based molecular markers utilized in the current study could be widely used in the future for screening and identifying wheat-*Ae*. *comosa* Robertsonian translocations.

Tiwari *et al*. developed molecular markers specific for *Ae*. *geniculata* chromosome 5M^g^S, and concluded that these markers may be useful for monitoring introgression into wheat from *Ae*. *comosa*, *Ae*. *geniculata* and *Ae*. *biuncialis* due to the fact that these *Aegilops* species share a common M genome^23^. However, the sequence-specific amplified polymorphic markers developed for the M-genome chromosomes of *Ae*. *comosa*^24^ seem not to completely support the previous conclusion. Furthermore, Molnár *et al*. found that some markers which were assigned to the M-genome chromosomes of *Aegilops* showed different chromosomal locations in the allopolyploid species^25^. In this research, primers TNAC1294 and TNAC1254 of homoeologous group 3 amplified the same polymorphic bands from wheat lines carrying the 3M and 5M chromosomes (Table 2). Moreover, primers TNAC1396, TNAC1496, TNAC1823, TNAC1888, TNAC1889, TNAC1902 and TNAC1884 were found to amplify polymorphic amplicons in M-genome chromosomes of *Ae*. *comosa* but could not generate polymorphisms for M^g^-chromosomes of *Ae*. *geniculata* (Table 2). Possible explanations for this are, gene duplication^25^ or chromosomal rearrangement^26^ might have occurred to M chromosomes in the process of forming polyploids. These results also imply that careful validation need to be done before applying the PLUG markers developed herein to other M-chromosome containing species.

Friebe *et al*. established the standard karyotype of *Ae*. *comosa*^27^, however, the C-banding patterns of different subspecies of *Ae*. *comosa* differed markedly. A similar observation was reported by Teoh *et al*.^28^. Later, FISH markers of *Ae*. *comosa* chromosomes using SSR probes ((ACG)_n_ and (GAA)_n_)^29^ and repeated DNA probes (pSc119.2, Afa family and pTa71)^30^ were developed. However, the FISH patterns of chromosomes 2M, 3M, 4M and 7M using pSc119.2 as a probe were different from those of this current research, and a similar phenomenon was also found by using (GAA)_n_ as a probe. Recently, Kwiatek *et al*. established the standard FISH patterns of *Ae*. *comosa* chromosomes using probes pAs1, pSc119.2, 5S and 35S rDNA^31^. However, the FISH patterns of chromosomes 2M, 4M and 7M using pSc119.2 as a probe were different from those of our present research. These FISH pattern differences mentioned above might be due to different origins of the accessions of *Ae*. *comosa*. Therefore a standard FISH pattern needs to be established for each individual research study when different accessions of *Ae*. *comosa* are used. Even though the FISH pattern using Oligo-pTa535-1 as a probe has not been reported earlier, it had been found that on its own it could not be used to identify the *Ae*. *comosa* chromosomes (Fig. 4B). The FISH patterns using the combined probes Oligo-pTa535-1 and Oligo-pSc119.2-1, or (GAA)_8_ could be used to distinguish the individual chromosome of *Ae*. *comosa* used in this research.

### Agronomical traits influences by introducing *Ae*. *comosa* chromosomes into wheat

Transferring alien chromosomes into wheat might lead to the change of disease resistance^13,14,32^, plant height^14,32,33^, leaf or spike morphology^14,32,34^, thousand kernel weight or seed hardness^14,32,35,36^, fertility^34,37^ and quality^14,32,35,38^. In our current research, the introduction of chromosomes 2M to 7M into wheat not only affected the spike morphology (Fig. 5), but also affected fertility of the apical spikelet (Fig. 5), resistances (Table 3), thousand kernel weight or tiller number. (Fig. 6). Therefore, a comprehensive characterization needs to be done for each individual addition or substitution line before embarking on chromosome engineering activities with the intention of recombining that alien chromosome with wheat. CS-*Ae*. *comosa* 6M addition showed a longer but elongated spike compared to CS, however, the CS-*Ae*. *comosa* 6M(6A) substitution line showed a shorter and dense spike, indicating that (a) chromosome 6M of *Ae*. *comosa* might possess gene(s) that affect longer spike, a similar phenomenon found in *Agropyron cristatum*^39^, (b) chromosome 6A of wheat might possess a compactness gene(s), a conclusion supported by ref.^40^, and (c) chromosome 6A of wheat may carry genes that affect spike length, supported by findings of ref.^41^. Based on these conclusions, we suggest the use of wheat-alien species chromosome introgression lines, including addition, substitution, translocation or deletions, to locate important functional genes on wheat or alien species chromosomes. Furthermore, the possibility exists that future researchers might narrow down the target region or even clone the genes that affect compactness or spike length from chromosome 6A using CS deletion lines and the reference sequences of CS.

### *Ae*. *comosa* chromosomes possess stripe rust and powdery mildew resistance new genes

Riley *et al*. found that chromosome 2M of *Ae*. *comosa* introduced into wheat improved stripe rust resistance^42^. Later, using induced homeologous pairing and crossing over, they transferred stripe rust resistance gene *Yr8*^2^and stem rust resistance gene *Sr34*^4,5^ from *Ae*. *comosa* into wheat. In our current research, the CS-*Ae*. *comosa* 2M addition line was nearly immune to stripe rust but this may not be attributable to gene *Yr8* because this gene has already lost its resistance in China^43^. Therefore, there might be another stripe rust resistance gene(s) on the chromosome 2M of *Ae*. *comosa* which needs further research into pathotype reactions. So far, a total of 60 powdery mildew resistance genes have been designated. Among them, 19 originate from wheat’s alien relatives, such as *Pm7*, *Pm8*, *Pm17* and *Pm20* from *Secale cereale*, *Pm12*, *Pm32* and *Pm53* from *Ae*. *speltoides*, *Pm13* from *Ae*. *longissima*, *Pm21* and *Pm55* from *Dasypyrum villosum*, *Pm19*, *Pm34*, *Pm35* and *Pm58* from *Ae*. *tauschii*, *Pm29* from *Ae*. *ovata*, *Pm40* and *Pm43* from *Thinopyrum intermedium*, *Pm51* from *Th*. *ponticum*, and finally *Pm57* from *Ae*. *searsii*. However, no powdery mildew resistance genes have so far been reported to have been transferred from *Ae*. *comosa* to wheat. In this research, we found that the CS-*Ae*. *comosa* 7M addition line was nearly immune to powdery mildew while wheat control CS was highly susceptible, indicating that chromosome 7M of *Ae*. *comosa* possesses a potentially new powdery mildew resistance gene(s). Both CS-*Ae*. *comosa* 2M and 7M additions are worthy of further study for production of chromosome translocations and then subsequent incorporation of their disease resistance into wheat breeding programs in China.

## Conclusion

In conclusion, we characterized, identified and evaluated a set of wheat-*Ae*. *comosa* chromosome lines, including 2M–7M addition lines and a 6M(6A) substitution line, using the newly developed *Ae*. *comosa* chromosome specific molecular markers and cytogenetic markers, disease resistance tests and agronomical traits investigations. Chromosomes 2M to 7M of *Ae*. *comosa* are stripe rust and powdery mildew resistance source for wheat breeding, respectively. However, the selection of plants with relatively negative effects should be avoided when inducing wheat-*A*. *comosa* chromosome translocations using chromosome engineering procedures.

## Methods

### Plant materials

*Triticum aestivum* cv. Jinan17 (JN17) and Jimai22 (JM22) cultivars were developed at the Crop Research Institute at Jinan, China. *T*. *aestivum* cv. Chinese Spring (CS) was provided by Prof. Zujun Yang, School of Life Science and Technology, University of Electronic Science and Technology of China, Chengdu. *Ae*. *comosa* (TA1967), a *T*. *turgidum-Ae*. *comosa* amphiploid (TA3402), and CS-*Ae*. *geniculata* 1M^g^, 2M^g^ and 7M^g^ addition lines (TA7655-TA7661) were provided by Mr. J. Raupp, Wheat Genetic and Genomic Resources Center, Kansas State University, USA. Six CS-*Ae*. *comosa* addition lines and one substitution line, tentatively named as wheat-*Ae*. *comosa* 2M–7M additions and a 6M(6A) substitution, were provided by Prof. S.M. Reader, John Innes Centre, UK.

### Disease resistance testing

Stripe rust, leaf rust, stem rust and powdery mildew resistance reactions of the suspected CS-*Ae*. *comosa* 2M–7M addition lines and the 6M(6A) substitution line and CS were tested. CS is highly susceptible to all four pathogens, hence the disease response scoring did not begin until CS was fully infected. Pathogenic race selection and disease response rating scale of four diseases were all according to ref.^14^. The pathogen inoculation methods of stripe rust, leaf rust and powdery mildew were according to ref.^44^, while stem rust inoculation was according to ref.^45^. Stripe rust resistance was determined on adult plants using isolates of races CY32, CY33 and Su-4 in the experimental farmland of School of Life Science and Technology, University of Electronic Science and Technology of China. Stem rust resistance was determined on seedlings using mixed isolates of 34MKGQM and 21C3CTHSM in the greenhouse of College of Plant Protection, Shenyang Agricultural University. Leaf rust resistance was determined on seedlings using mixed leaf rust isolates of THTT, PHTT, THKS, THTS and THKT in the greenhouse of College of Plant Protection, Agricultural University of Hebei. Powdery mildew resistance was determined on both seedlings (in greenhouse) and adult plants (field) following inoculation with mixed powdery mildew races collected from four different cities including Jinan, Linyi, Dezhou and Heze of Shandong Province.

### Agronomical trait investigation

Thirty individual plants of the suspected CS-*Ae*. *comosa* 2M–7M additions, 6M(6A) substitution and CS were planted in farmland at four different cities including Jinan, Dezhou, Heze and Linyi of Shandong Province on October 25, 2015. The experimental design, data collection of plant height, spike length, flag leaf length and width, tiller number, spikelet number, grain number per 30 spikes and thousand-kernel weight were according to ref.^14^.

### Data processing and qualification

Data on the number of tillers per plant from Jinan was not obtainable. Data processing and *t*-test was performed using Microsoft Excel 2010. The data from four sites (tiller number, across the three cities) were completely consistent with each other, and the trait variation when compared to the background genotype CS will be regarded as attributable to the presence of the alien chromatin. Alternatively, it might be considered as a result of interaction of genotype and environments. In this research, only the former will be discussed.

### DNA isolation and PLUG-PCR

Total genomic DNA was prepared from young leaves using the SDS protocol^46^. A total of 526 PLUG primer pairs were synthesized according to refs^47,48^, of which 57, 67, 85, 71, 78, 59 and 109 pairs belonged to chromosome homoeologous groups 1 to 7, respectively. All primer pairs were synthesized by Chengdu Ruixin Biological Technology Co., Ltd., and PCR protocol followed that according to refs^47,48^. In order to obtain high levels of polymorphism, the PCR products were digested with the 4-base cutter enzymes *Hae*III and *Taq*I according to refs^47,48^ and were separated on 2% agarose gels.

### Fluorescence *in situ* hybridization (FISH) analysis

Root tip treatments and chromosome slide preparations were according to ref.^49^. Probes Oligo-pTa535-1, Oligo-pSc119.2-1 and (GAA)_8_ were synthesized by Chengdu Ruixin Biological Technology Co., Ltd. Probe sequences, the fluorochromes for probe labeling, FISH protocols and labeled DNA signal detection methods were according to refs^50,51^. FISH using (GAA)_8_ as a probe could be used to identify all 42 wheat chromosomes except 1A, 3D, 4D, 5D and 6D, as described by ref.^50^. FISH using Oligo-pSc119.2-1 and Oligo-pTa535-1 probes could identify all 42 wheat chromosomes simultaneously as described by ref.^51^. Photomicrographs of FISH chromosomes were taken using an Olympus BX-51 microscope.

## Supplementary information

Supplementary figures
