# Supplementary material for: Characterization, identification and evaluation of a set of wheat-Aegilops comosa chromosome lines
Source: Sci Rep. 2019 Mar 18;9:4773. doi: 10.1038/s41598-019-41219-9 (PMC6423130; doi:10.1038/s41598-019-41219-9)
Supplement: Supplementary file 1 — Supplementary figures [file 41598_2019_41219_MOESM1_ESM.pdf]

Characterization, identification and evaluation of a set of wheat-*Aegilops comosa* chromosome lines

Cheng Liu [1,2], Wenping Gong [1], Ran Han[1], Jun Guo [1], Guangrong Li [3], Haosheng Li [1], Jianmin Song [1,2], Aifeng Liu [1], Xinyou Cao [1,2], Shengnan Zhai [1], Dungong Cheng [1], Genying Li [1,2], Zhendong Zhao [1], Zujun Yang [3], Jianjun Liu [1]* and Stephen M Reader [4]*

[1] Crop Research Institute, Shandong Academy of Agricultural Sciences/Key Laboratory of Wheat Biology and Genetic Improvement in the North Yellow & Huai River Valley, Ministry of Agriculture/National Engineering Laboratory for Wheat & Maize, Jinan 250100, China; [2] Colloge of Life Science, Shandong Normal University, Jinan 250014, China

[3] School of Life Science and Technology, University of Electronic Science and Technology of China, Chengdu 610054, China; [4] John Innes Centre, Norwich Research Park, Colney, Norwich, NR4 7UH, UK;

* Correspondence: ljjsaas@163.com; etreaders@gmail.com; Tel.:+86-531-6665-9561; Fax: +86-531-9476

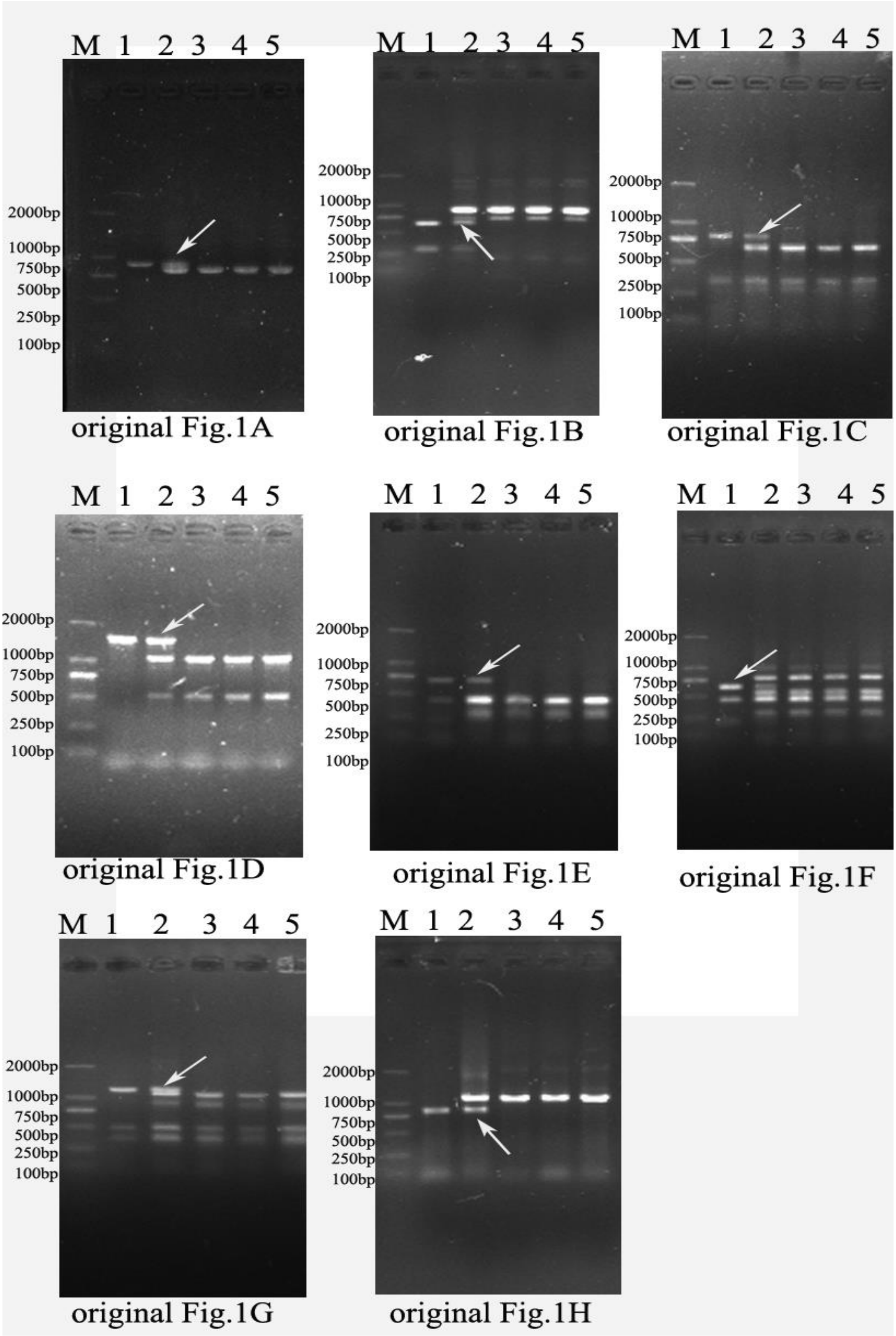

Original Fig. 1. PCR patterns of primer pairs TNAC1204 (A), TNAC1137 (B), TNAC1329 (C), TNAC1331 (D), TNAC1737 (E), TNAC1740 (F), TNAC1800 (G) and TNAC1924 (H).

Lane M indicates Marker DM2000. Lanes 1-5 in figures A-H are *Ae. comosa*, *T. turgidum-Ae. comosa* amphiploid, CS, JM22 and JN17, respectively.

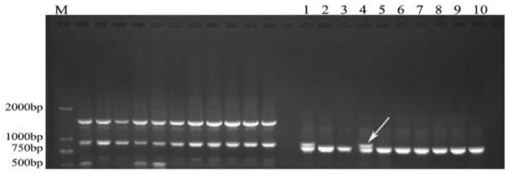

original Fig.2A

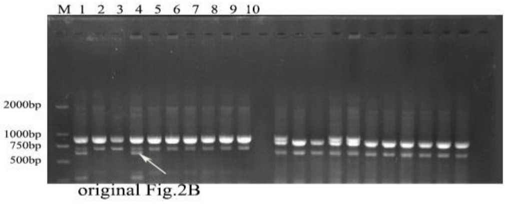

original Fig.2B

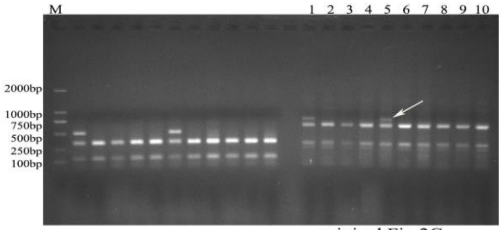

original Fig.2C

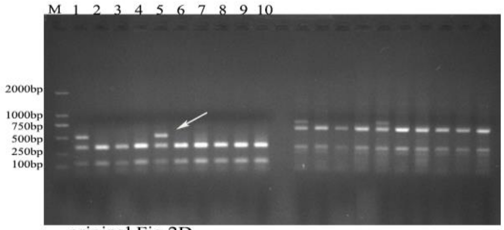

original Fig.2D

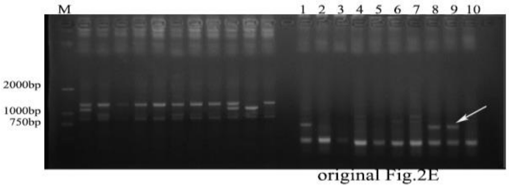

original Fig.2E

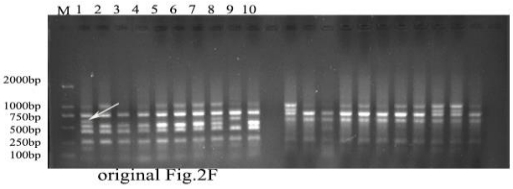

original Fig.2F

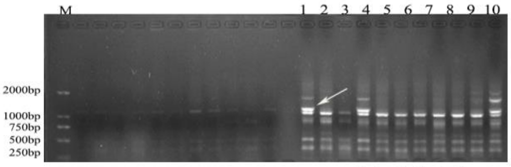

original Fig.2G

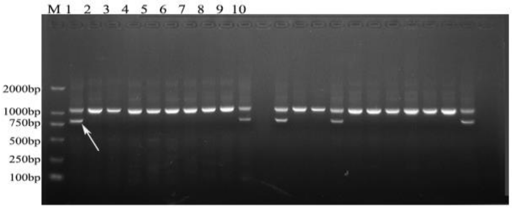

original Fig.2H

Original Fig. 2. PCR patterns of primer pair TNAC1204 (A), TNAC1137 (B), TNAC1329 (C), TNAC1331 (D), TNAC1737 (E), TNAC1740 (F), TNAC1800 (G) and TNAC1924 (H).

Lane M indicates Marker DM2000. Lanes 1-10 in figures A-H are *T. turgidum*-*Ae. comosa* amphiploid, CS, CS-*Ae. geniculata* $1M^g$ addition, CS-*Ae. comosa* 2M-6M additions, CS-*Ae. comosa* 6M(6A) substitution and CS-*Ae. comosa* 7M addition, respectively.
